# Passive Location Resource Scheduling Based on an Improved Genetic Algorithm

**DOI:** 10.3390/s18072093

**Published:** 2018-06-29

**Authors:** Jianjun Jiang, Jing Zhang, Lijia Zhang, Xiaomin Ran, Yanqun Tang

**Affiliations:** National Digital Switching System Engineering and Technological Research Center (NDSC), Zhengzhou 450000, China; zhangjingok@sina.com (J.Z.); vatzhang1993@163.com (L.Z.); rxmhkz@tom.com (X.R.); tangyanqun@126.com (Y.T.)

**Keywords:** passive location, NP-hard, scheduling, genetic algorithm, angle-of-arrival

## Abstract

With the development of science and technology, modern communication scenarios have put forward higher requirements for passive location technology. However, current location systems still use manual scheduling methods and cannot meet the current mission-intensive and widely-distributed scenarios, resulting in inefficient task completion. To address this issue, this paper proposes a method called multi-objective, multi-constraint and improved genetic algorithm-based scheduling (MMIGAS), contributing a centralized combinatorial optimization model with multiple objectives and multiple constraints and conceiving an improved genetic algorithm. First, we establish a basic mathematical framework based on the structure of a passive location system. Furthermore, to balance performance with respect to multiple measures and avoid low efficiency, we propose a multi-objective optimal function including location accuracy, completion rate and resource utilization. Moreover, to enhance its practicability, we formulate multiple constraints for frequency, resource capability and task cooperation. For model solving, we propose an improved genetic algorithm with better convergence speed and global optimization ability, by introducing constraint-proof initialization, a penalty function and a modified genetic operator. Simulations indicate the good astringency, steady time complexity and satisfactory location accuracy of MMIGAS. Moreover, compared with manual scheduling, MMIGAS can improve the efficiency while maintaining high location precision.

## 1. Introduction

Passive location plays a significant role in modern communication scenarios for its satisfactory concealment based on the passive reception pattern. In addition, with the characteristics of long distance measurement and high robustness [1], passive location techniques are of significance in both civil and military areas. The major passive location technique is angle-of-arrival (AOA) [2], which obtains the target location by combining the measured angles of each station and whose performance is highly affected by scheduling plans [3,4]. Resource scheduling is implemented to establish an optimized task-to-resource dispatch with the purpose of maximizing the total performance (such as location accuracy and task completion rate) and avoiding the conflicts effectively. However, despite its importance, little attention is paid to the scheduling mechanism. In recent years, studies in passive location have mainly focused on location algorithms [5,6], accuracy analysis [7], filters and tracking [8,9]. There is little information available in the literature about passive location resource scheduling. The scheduling method mostly used today, which we call traditional manual scheduling, is unable to handle the increasing number of tasks and overloaded resources, thus leading to the following serious problems.

- Low efficiency. Because manually allocated broadcast resources cannot achieve parallel processing very well, when task conflicts occur, some signals may be lost, reducing efficiency.
- Unstable location accuracy. Because location accuracy is highly relevant to the stations-to-tasks schedule plans, it is possible to encounter low location accuracy due to mistakes caused by human subjectivity.

To address these problems and achieve high efficiency and location accuracy, new intelligent automated scheduling methods urgently need to be studied. Xiu et al. [10] analyzed constellations in bearing-only location systems and proposed an optimal geometric relationship to improve location precision and minimize circular probable error, providing an important reference for station scheduling. Prasan et al. [11] proposed the pre-scheduling-based k-coverage group scheduling and self-organized k-coverage scheduling algorithms to address problems in existing sleep-scheduling algorithms.

Hu et al. [12] established a centralized framework for sensor scheduling in wireless sensor networks and modeled the problem using integer linear programming. In [13], the proposed algorithms, which deal directly with minimizing the mean squared error, are based on the convex relaxation approach to address the binary optimization scheduling problems that are formulated in sensor network scenarios. Xu et al. [3] analyzed the Cramer-Rao lower bound of AOA and discussed the measurement of location accuracy based on the information matrix. Wei et al. [9] proposed a modified binary particle swarm optimization (BPSO) algorithm motivated by the information matrix in the wireless sensor network nodes scheduling problem for tracking. Sun et al. [14] were inspired by Xu et al. and Wei et al. and proposed a BPSO algorithm motivated by geometric dilution of precision deduced from the information matrix for station scheduling. However, Sun et al. did not solve the problem thoroughly because they neglected to consider complex constraints and efficiency. Furthermore, previous studies seldom discuss scheduling strategies for scenes with multiple concurrent tasks. Therefore, to solve passive location station scheduling more specifically, the scheduling method must satisfy these conditions. (1) The optimal objective must involve not only accuracy but also other important factors, especially efficiency. (2) Complex constraints are formulated. (3) The method can support multiple concurrent task scenarios.

Inspired by the factors mentioned above, we propose a new method called multi-object, multi-constraint and improved genetic algorithm-based Scheduling (MMIGAS), enhancing the efficiency and practicability for passive location resource scheduling. Our first contribution is to construct a mathematical primary centralized optimization model of passive location resource scheduling according to a practical system framework. Second, we introduce position dilution of precision (PDOP) to measure the location accuracy and define other objective values, such as completion rate, resource utilization and task priority. These values are combined into a multi-objective function to ensure that our schedule can balance performance with respect to multiple aspects, specially efficiency. Third, based on several important assumptions, we formulate complex constraints in three categories: frequency, station capability and task cooperation requirement, thus improving practicability. Moreover, since the model is an NP-hard problem, we conceive an improved genetic algorithm (IGA) by introducing constraint-proof initialization (CI), penalty function (PF) and modified genetic operator (MGO), which can achieve a better convergence rate and global optimization ability. Simulations show that MMIGAS has a satisfactory astringency and stability of time complexity. Additionally, the method is appropriate for multiple concurrent targets. Compared with manual scheduling, MMIGAS can clearly improve the efficiency while maintaining high location accuracy.

The remainder of this paper is organized as follows. Section 2 introduces the primary framework of the location system, clarifies some critical assumptions and establishes a basic mathematical optimization model. In Section 3, we demonstrate our design for multiple objectives and multiple constraints. In Section 4, we illustrate the three steps of the IGA: CI, PF and MGO. Section 5 presents the experimental results and analyses the performance of proposed method. Section 6 concludes the paper.

## 2. Problem Formulation

**Figure 1:**
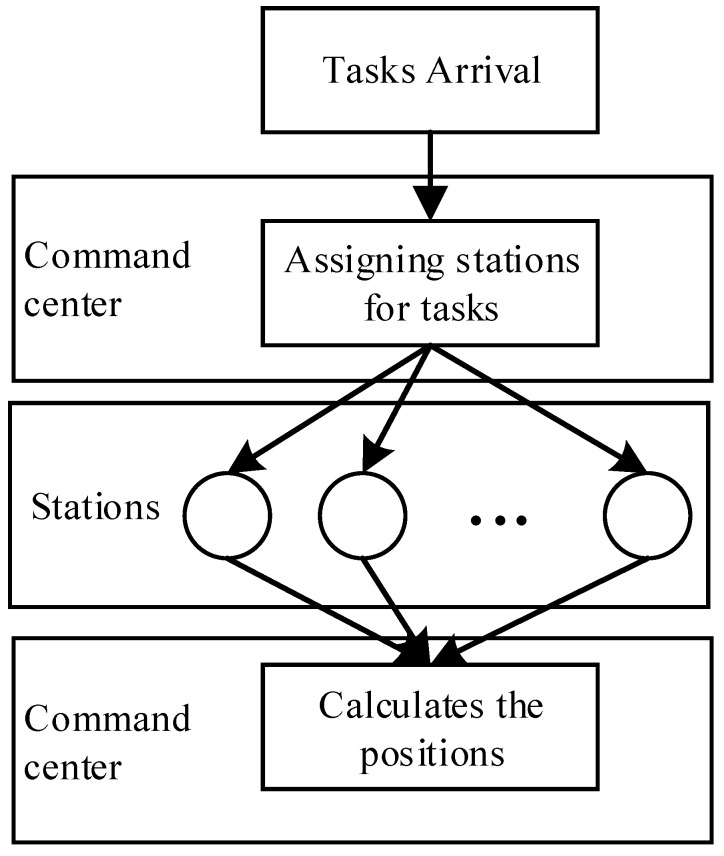
Procedure for passive location in a practical system.

The passive location channel is heavily crowded due to the narrowband and dense signals. The time-varying fading and silence zones also cause many problems, making the passive location problem a difficult task and topic of interest [15,16,17,18,19,20]. To increase the location accuracy and flexibility, a location system generally uses a cooperative location approach [21], which consists of the steps of task arrival, command downloading, data uploading and position output, as shown in Figure 1.

**Figure 2:**
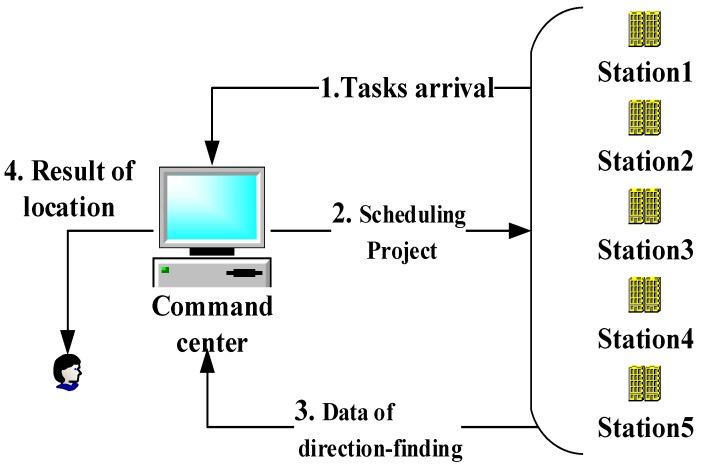
Architecture of a passive location system.

Moreover, the system has a centralized architecture consisting of the command center and direction-finding stations, as shown in Figure 2. Therefore, the essence of station scheduling is a centralized tasks-to-resources allocation, which guides us to establish a combination optimization model.

### 2.1. Assumptions and Scenario Declaration

Because the passive location scenario is extremely complicated due to complex channels [22], we make some critical assumptions to simplify this problem.

- We ignore the silence zone. This assumption is made to reduce the space constraint and make the model more concise. According to related studies, potential silence zones can be fully avoided if appropriate locations for building stations are chosen [10].
- Signals from all directions have the same gain in the receiving antennas. This assumption is made to ensure the strength of the signal directly reflects the distance between the station and the target, which simplifies the discussion about geographical relationships. This can be achieved by using an omnidirectional antenna.
- Targets are static or moving slowly enough that their locations do not noticeably change over a short time. This assumption avoids a heavy time delay or even invalidation of the scheduling due to drastic changes in geographical relationships.
- Prior knowledge about targets is available. This assumption ensures that the approximate potential area of the target is known, which is an essential condition of using the proposed scheduling method.
- In addition, the scenario discussed in this paper is specified by the following declarations.
- We only discuss the scheduling activity in one slot. We define one slot as the duration of a scheduling and location process. It is the minimum interval needed to implement one complete activity in the passive location system. Therefore, any length of time can be considered to be a sum of several slots.
- We only discuss narrow-band stations because wide-band stations can be considered the sum of several different narrow-band stations.

### 2.2. Notation

Stations and signals are considered to be resources and tasks, respectively. And to demonstrate their allocation relationship, we structure a scheduling matrix as follows.

1. Task: We denote tasks as $T=\left\{t_{1},t_{2},\ldots ,t_{i},\ldots t_{N_{m}}\right\}$, where $N_{m}$ is the total number of tasks. We use four attributions to describe a task: frequency, location, collaboration and priority, $\forall t_{i}\in T,t_{i}=\left\{{\hat{Z}}_{i},{\hat{\lambda }}_{i},{\hat{B}}_{i},{\hat{H}}_{i}\right\}$, where i denotes the task sequence number.
  - ${\hat{Z}}_{i}=\left[{\hat{f}}_{\min}\left(i\right),{\hat{f}}_{\max}\left(i\right)\right]$ gives the frequency range of the task signal, where ${\hat{f}}_{\min}\left(i\right)\in R,{\hat{f}}_{\max}\left(i\right)\in R$ and ${\hat{f}}_{\min}\left(i\right)\leq {\hat{f}}_{\max}\left(i\right)$.
  - ${\hat{\lambda }}_{i}=\left({\hat{x}}_{i},{\hat{y}}_{i}\right)$ denotes the two-dimensional coordinates of task i, where ${\hat{x}}_{i}\in R,{\hat{y}}_{i}\in R$.
  - ${\hat{B}}_{i}$ gives the cooperative station number requirement for task location, where ${\hat{B}}_{i}\in Z^{+}$.
  - ${\hat{H}}_{i}\in \left\{1,\ldots ,6\right\}$. We define the priority of tasks as an integer ranging from 1 to 6. The task is more important if its ${\hat{H}}_{i}$ is higher.
2. Resources: We denote $N_{s}$ direction-finding stations as $K=\left\{k_{1},k_{2},\ldots ,k_{j},\ldots ,k_{N_{s}}\right\}$. We use three attributions to describe one station (resource): frequency, location and capability, $\forall k_{j}\in K,k_{j}=\left\{Z_{j},\lambda_{j},C_{j}\right\}$. Here, j denotes the station sequence number.
  - $Z_{j}=\left[f_{\min}\left(j\right),f_{\max}\left(j\right)\right]$ is the frequency range at which the station implements direction-finding, where $f_{\min}\left(j\right)\in R,f_{\max}\left(j\right)\in R$ and $f_{\min}\left(j\right)\leq f_{\max}\left(j\right)$.
  - $\lambda_{j}=\left(x_{j},y_{j}\right)$ denotes the two-dimensional coordinates of station j, $x_{j}\in R,y_{j}\in R$.
  - $C_{j}$ is the maximum number of tasks the station can process synchronously, implying its working capacity, where $C_{j}\in Z^{+}$.
3. Scheduling matrix: We design a binary solution matrix with $N_{s}$ columns and $N_{m}$ rows as $A={\left[a_{ij}\right]}_{N_{m}\times N_{s}}$, where $a_{ij}$ indicates the relation between station j and task i. Here $a_{ij}=1$ means that station j is assigned to process task i, while $a_{ij}=0$ means j is not assigned to process i.

### 2.3. Optimization Model

Based on the analysis mentioned above, we formulate the passive location resource scheduling problem as a combination optimization problem. Denote F as the objective function, $h_{i}$ as the equality constraints and $g_{i}$ as the inequality constraints. Then, the mathematical model is:

(1) $$\{\begin{array}{ll} & \max\left\{F\left\{A\right\}\right\}\\ s.t. & h_{i}\left\{A\right\}=0,\;i=1,2,3\ldots \\ & g_{i}\left\{A\right\}\geq 0,\;j=1,2,3\ldots \end{array}$$

## 3. Multi-Objective and Multi-Constraint Properties

### 3.1. Objective Function

To balance different aspects of the performance and obtain a comprehensive solution, we design a multi-objective function using a linear weighting as follows:

(2) $$F=\sum_{q}\varphi_{q}\omega_{q}$$

Here $\varphi_{q}$ is the objective and $\omega_{q}$ is the weight. The definition and calculation of objectives and weights is illustrated in the following part of this section.

#### 3.1.1. Location Accuracy

Because the fundamental requirement of passive location resource scheduling is to obtain a scheduling plan with a satisfactory location accuracy, the objective function must involve the location accuracy. We introduce PDOP as the measure of location accuracy. PDOP quantifies the geographical relationships between receivers and targets, which can directly impact the performance of the location activity. Smaller PDOP means better performance and on the contrary, larger PDOP means worse performance. Based on [14,23], PDOP is calculated as follows:

(3) $$p_{i}=\frac{{\left\{\sum_{j-1}^{M_{1}}\frac{1}{r_{j}{}^{2}}\frac{1}{{\left(\sigma_{\phi }^{}\left(j\right)\right)}^{2}}\right\}}^{\frac{1}{2}}}{\det{\left(L_{i}\right)}^{\frac{1}{2}}}$$

(4) $$\det\left(L_{i}\right)=\sum_{\left\{u,v\right\}\in M_{2}}\frac{1}{{\left(\sigma_{\phi }^{}\left(u\right)\right)}^{2}{\left(\sigma_{\phi }^{}\left(v\right)\right)}^{2}}\frac{\sin^{2}\left(\phi_{u}-\phi_{v}\right)}{r_{u}{}^{2}r_{v}{}^{2}}$$

Here, $p_{i}$ is the PDOP value of task i, $L_{i}$ is the Fisher information matrix, $r_{j}$ is the distance from station j to the target, $\phi_{j}$ is the direction angle, $\sigma_{\phi }^{}\left(j\right)$ is the measurement error of $\phi_{j}$, $M_{1}$ denotes the number of stations that participate in the task and $M_{2}$ denotes the set of every match of two stations in $M_{1}$. For example, when $M_{1}=\left\{1,2,3\right\}$, then $M_{2}=\left\{\left\{1,2\right\},\left\{2,3\right\},\left\{1,3\right\}\right\}$. To make the objective factor positive with respect to location accuracy, we formulate $\varphi_{p}$ as the reciprocal of the sum of PDOP as:

(5) $$\varphi_{p}=\frac{1}{\sum_{i}p_{i}}$$

Additionally, we set $\omega_{p}=1$ and the objective function is:

(6) $$F=\varphi_{p}+\sum_{q}\varphi_{q}\omega_{q}$$

#### 3.1.2. Completion Rate

To avoid the task missing problem and low efficiency, we define an objective factor named completion rate $\varphi_{1}$ which is equal to the ratio of completed tasks n to the total number of tasks $N_{m}$.

(7) $$\varphi_{1}=\frac{n}{N_{m}}$$

Weight $\omega_{q}$ is calculated as the product of scaling factor $\eta_{q}$ and concern level $c_{q}$.

(8) $$\omega_{q}=\eta_{q}c_{q}$$

Here, $\eta_{q}$ transforms objective factor $\omega_{q}$ to the same order of magnitude of $\varphi_{p}$, which avoid poor performance of F caused by the wrong size of $\varphi_{p}$. It helps F to reflect various factors evenly. Objective factor $\eta_{i}$ is calculated as follows:

(9) $$\eta_{i}=10^{\log_{10}\left\lfloor \frac{\varphi_{p}}{\varphi_{i}}\right\rfloor }$$

Here ⌊x⌋ means the maximum integer value less than x and $c_{q}$ indicates a user’s concern about the objective factor, which is shown as Equation (10).

(10) $$\{s.t.\begin{array}{c} c_{q}\in \left[0,1\right]\\ \sum_{q}c_{q}=1 \end{array}$$

#### 3.1.3. Resource Utilization

To prevent resource waste, we introduce the resource utilization in Equation (11), which is the ratio of occupied resources m to the total number of resources $N_{s}$.

(11) $$\varphi_{2}=\frac{m}{N_{s}}$$

Analogously, weight $\omega_{2}$ is calculated by (8)–(10).

#### 3.1.4. Task Priority

Inspired by priority scheduling [24], we design objective factor $\varphi_{3}$ based on task priority, which makes the model prefer the task with the highest priority, shown in Equation (12).

(12) $$\varphi_{3}=\sum_{i}{\hat{H}}_{i}$$

Here, $\varphi_{3}$ equals the sum of priorities of completed tasks. As above, weight $\omega_{3}$ is analogously calculated by (8)–(10).

In conclusion, the formulation of multi-objective function F is as follows:

(13) $$F=\varphi_{p}+\sum_{q-1}^{3}\varphi_{q}\omega_{q}=\varphi_{p}+\sum_{q-1}^{3}\varphi_{q}\eta_{q}c_{q}$$

### 3.2. Constraints

Due to the complexity of wireless communication channels [25], the resource scheduling problem, involving many constraints on time, space, frequency and other factors, need to be analyzed and formulated to ensure our mathematical model is reasonable. Based on our assumptions, we ignore the constraints in the time and space domains and simplify the complex conditions into three categories: frequency, station capability and task cooperation requirements.

#### 3.2.1. Frequency

The direction-finding activity cannot be implemented when the frequency of the task is beyond the station’s range. We formulate such a constraint as follows:

(14) $$\forall t_{i}\in T,\forall k_{j}\in K,\{\begin{array}{c} a_{ij}\left({\hat{f}}_{\min}\left(i\right)-f_{\min}\left(j\right)\right)\geq 0\\ a_{ij}\left(f_{\max}\left(j\right)-{\hat{f}}_{\max}\left(i\right)\right)\geq 0 \end{array}$$

#### 3.2.2. Station Capability

For narrow-band stations, receivers are highly restricted, leading to the limitation of the number of tasks that can be implemented by one station synchronously.

(15) $$\forall j\in \left[1,N_{s}\right],\sum_{i-1}^{N_{S}}a_{ij}\leq C_{j}$$

#### 3.2.3. Task Cooperation Requirement

Because different tasks may have different cooperation requirements, a sufficient number of stations should be allocated to the task.

(16) $$\forall i\in \left[1,N_{t}\right],\sum_{j-1}^{N_{s}}a_{ij}\geq {\hat{B}}_{i}$$

## 4. IGA

Due to the strong relevant effects among tasks and resources, the proposed model becomes an NP-hard problem [26], for which a genetic algorithm (GA) is suitable for searching for a solution because of its adaptive global optimization ability. Originally, a GA was conceived based on the simulation of heredity and evolution in the natural environment [27].

**Figure 3:**
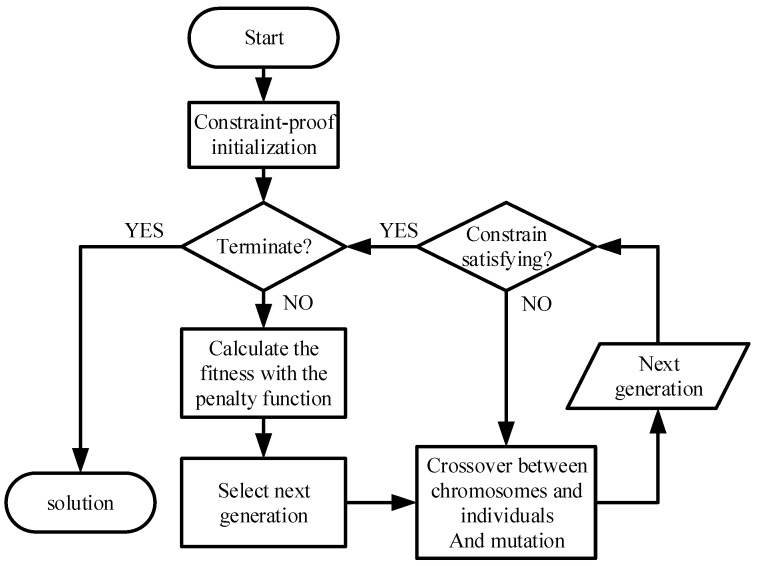
Procedure for the Improved Genetic Algorithm (IGA).

In the algorithm initialization, various initial feasible solutions are randomly generated as the original genetic group. During the iterations, genetic operators construct the offspring based on the parental genetic group, where the genes with highest environmental fitness can survive and multiply, thus conveying the dominated characteristics into the final generation. Eventually, the algorithm obtains an optimal solution. Compared with other algorithms, GA has low complexity and high practicability [28], thus being appropriate for the passive location resource scheduling problem. However, the normal GA has the disadvantages of a low convergence rate and local optimization dilemma. To make it more suitable for our application scenario, we make several improvements by implementing CI, PF and MGO, helping the algorithm to obtain a satisfying solution quickly. The objective function is utilized as the fitness function, while the decision matrix is used for gene construction. The process of the algorithm is shown in Figure 3 and details in Algorithm 1.

**Algorithm 1. IGA**.1. Parameters setting and constraint-proof initialization.2. Terminate? Yes→Step 6, No→Step3.3. Calculate the fitness with the PF and select next generation.4. Use MGO to get next generation.5. Constrain satisfaction? Yes→step 2, No→Step 4.6. Get the solution.

### 4.1. CI

**Figure 4:**
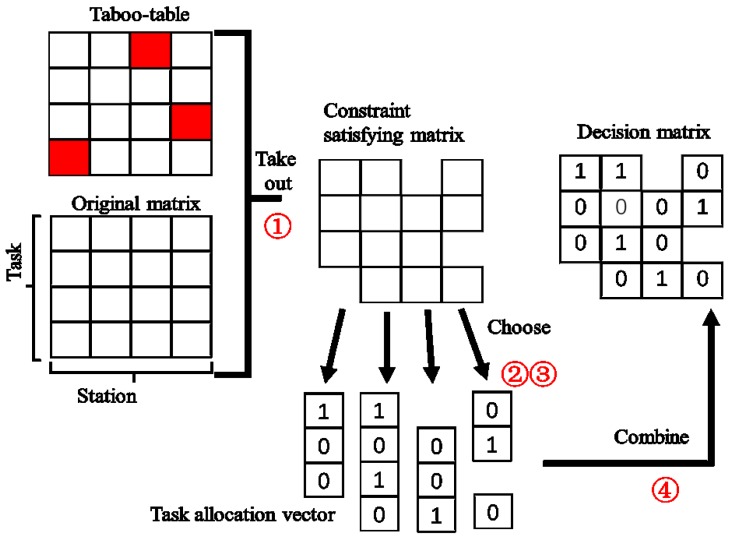
Procedure for constraint-proof initialization (CI).

Traditional GA is very sensitive to the initial values [29]. When the initial values do not meet the constraints, the algorithm is unable to calculate the fitness and thus the evolution direction is unclear, which substantially reduces the convergence rate and even leads to the non-convergence. Therefore, in order to provide a specific evolution direction at the beginning and improve the astringency, we implement the CI by building a taboo-table which marks out the element unable to satisfy the frequency constrain and coding the scheduling matrix stepwise. The process is shown in Figure 4.

Step ①: Generate the original matrix according to the number of tasks and stations. Build up a taboo-table by using Equations (14)–(16). And take away the taboo-table from the original matrix to obtain the frequency-satisfying matrix.

Step ②: Select one column of the matrix as a vector, which indicates the task scheduling table of one station. Value it randomly within the capability of the resource and then use it as a task allocation vector. 

Step ③: Repeat Step ② for every column in the frequency-satisfying matrix to obtain the task allocation vectors of all stations.

Step ④: Combine the task allocation vectors as a decision matrix.

### 4.2. PF

To enhance the algorithm performance, the original genetic group must satisfy all constraints in our model. CI guarantees the original genetic group will meet the constraints on frequency and resource capability. For the task cooperation constraint, we introduce the PF method based on Section 2.2 and Equation (1).

Denote Ω as the penalty factor. Set $\Omega >1,\Omega \in Z^{+}$. Define $X_{i}=\sum_{j-1}^{N_{s}}a_{ij}$ as the number of stations scheduled for task i. The PF is denoted as P:

(17) $$P=\sum_{i-1}^{N_{m}}\Omega^{\left(B_{i}-X_{i}\right)}$$

Therefore, the fitness function becomes:

(18) Fitness=F−P

According to (17) and (18), if one solution does not meet the task cooperation requirement, its P will increase exponentially, rapidly reducing its fitness and causing it to be eliminated during the iteration. Therefore, solutions eventually that survive must satisfy the task cooperation constraint.

### 4.3. MGO

The preceding section introduces the generation of the initial solution and the penalty function. Then, we will introduce the modified genetic operation of the solution which contains roulette wheel, elitism selection, crossover and mutation.

#### 4.3.1. Roulette Wheel and Elitism Selection

We introduce a combined roulette-wheel and elitism selection strategy, which can keep the randomness of offspring and prevent the loss of the best individual in the group. First, the individuals with the highest fitness are directly saved to the next generation. Second, parents are selected according to probability $p_{g}$, of individual l, which is calculated as follows:

(19) $$p_{g}^{}\left(l\right)=\frac{Fitness\left(l\right)}{\sum_{j-1}^{N_{p}}Fitness\left(j\right)}$$

where $N_{p}$ is the population of genetic group in this algorithm and Fitness(l) is the fitness of individual l.

#### 4.3.2. Crossover

1. Crossover between chromosomes

**Figure 5:**
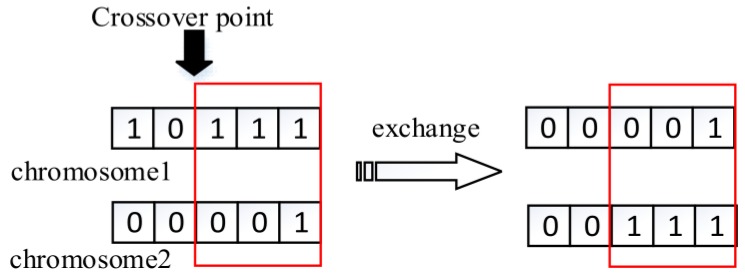
Individual crossover.

There is a probability $p_{cc}$ that chromosome crossover will happen for each individual. In the case, two chromosomes will be randomly selected and exchange their gene in the same location. Parameters $p_{cc}$ is set according to human experience, normally ranging from 0.3 to 0.7. Figure 5 shows the crossover process.

2. Crossover between individuals

**Figure 6:**
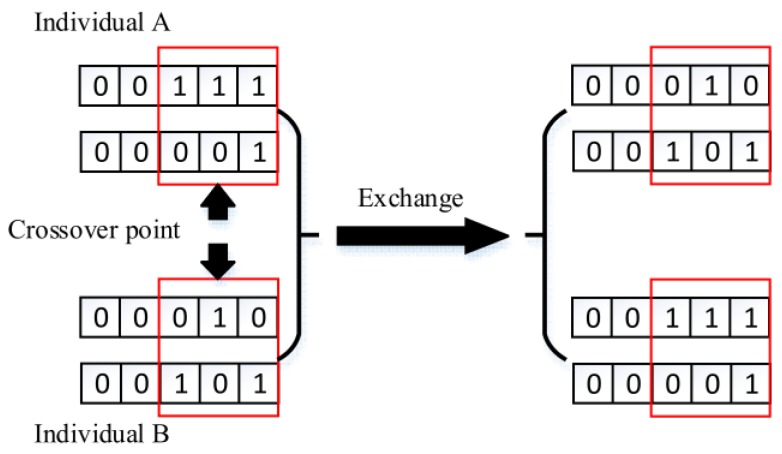
Chromosome crossover.

In the same way, we need to consider the concurrent processing of multiple tasks. So, there is a probability $p_{ci}$ for individual crossover among the entire group, which is the operation that one individual exchanges part of its genes with another. The location at which the gene exchange happens is selected randomly. Probability $p_{ci}$ is set by experience as well, normally ranging from 0.3 to 0.7. Figure 6 shows the process of two concurrent tasks.

3. Mutation

**Figure 7:**
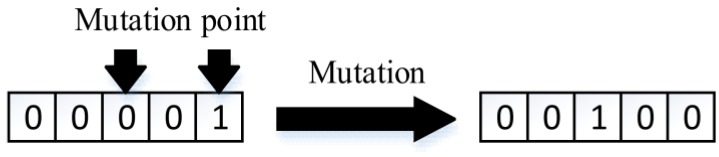
Mutation.

In every individual, there is a probability $p_{m}$ of mutation, which is when one or more genes inside this individual change themselves randomly. Parameter $p_{m}$ usually ranges from 0.01 to 0.1. An example of mutation is shown in Figure 7.

## 5. Experiment Results and Discussion

### 5.1. Simulation Setting

We simulate a passive location scenario based on AOA. The exact location, frequency and capability of stations are all known. Moreover, the approximate area, communication frequency, priority and cooperation requirement of tasks are known as well. Note that we only discuss the one-slot and narrow-band condition. Scenario and algorithm parameters are shown in Table 1, Table 2 and Table 3. First, we mainly consider the location accuracy and completion rate, so in this experiment objective function weights are set as $c_{1}=1$ and $c_{2}=c_{3}=0$. In addition, chromosome crossover probability, individual crossover probability and mutation probability are set according to Section 4.3. The direction angle error associated with the positioning accuracy is generated according to N(0,1) Gaussian distribution and the penalty factor is Ω=5. Moreover, the population number of chromosomes is 10 and the max iteration of genetic algorithm is 2000.

The stations and task signals were randomly distributed in a 50 km × 50 km area. The frequency range of stations and tasks were decided by their beginning frequencies and bands. The capabilities are randomly varied between 1 and 2, while the cooperation requirements are randomly varied between 2 and 3. And the locations of stations and task signals are randomly generated according to uniform distribution.

### 5.2. Experiment Results

**Figure 8:**
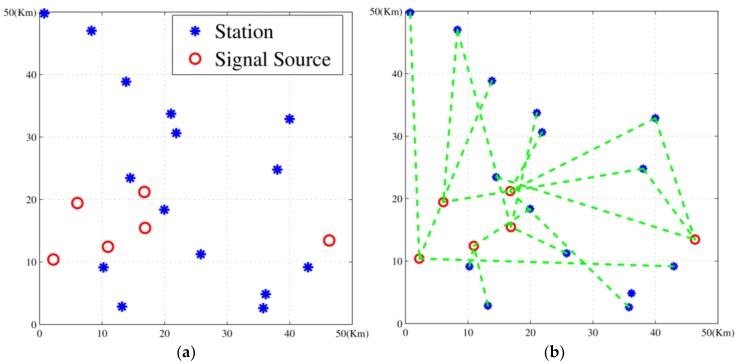
Passive location resource scheduling diagram with 15 stations and six tasks: (**a**) distribution of resources and tasks; (**b**) the scheduling plan: dotted green line represents task assignment.

We built up a scenario with 15 stations and six tasks. The solution given by the proposed algorithm is shown in Figure 8 and Table 4. The result indicates that the method can work out an appropriate solution and handle the NP-hard problem effectively.

**Figure 9:**
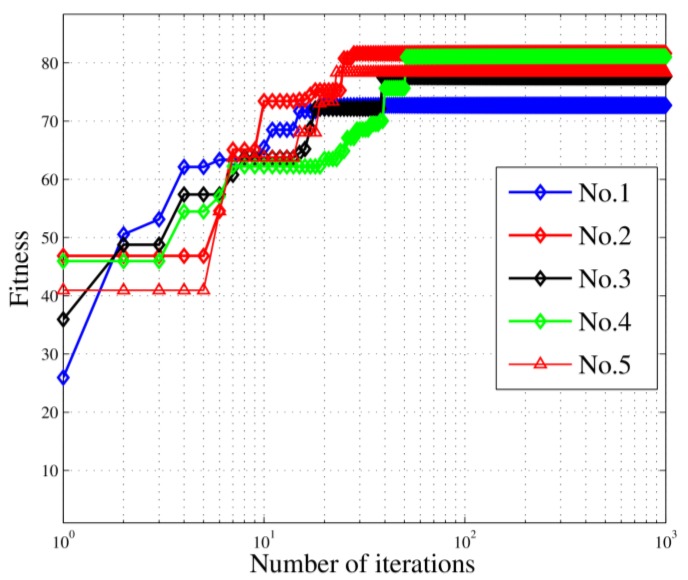
Convergence curves in the Monte-Carlo experiment.

Furthermore, we ran a Monte-Carlo experiment by repeating this simulation 300 times to examine the astringency. To better show the effect of the algorithm, the 300 experiments were divided into five groups (No. 1~No. 5), where each group including 60 experiment results and each curve representing the average value of each group. In each experiment, the location of the targets and the location of the stations are randomly generated. The convergence curves are shown in Figure 9, illustrating that the convergence values are very close to one another. This method can enlarge the search space and help the algorithm jump out of local minima. Moreover, all the curves reached their convergence in less than 100 iterations, showing the rapid convergence which means that the algorithm can finish calculation in a short time. Such a characteristic is very important for the passive location problem.

### 5.3. Results and Discussion

The experimental results show that the proposed algorithm can solve the multi-objective and multi-constraint scheduling problems. To further verify the performance of the IGA and analyze the experimental results, we analyze the performance and indicators, including the performance of the IGA and some experiment indicators, such as time complexity and location accuracy.

#### 5.3.1. Performance of the IGA

**Figure 10:**
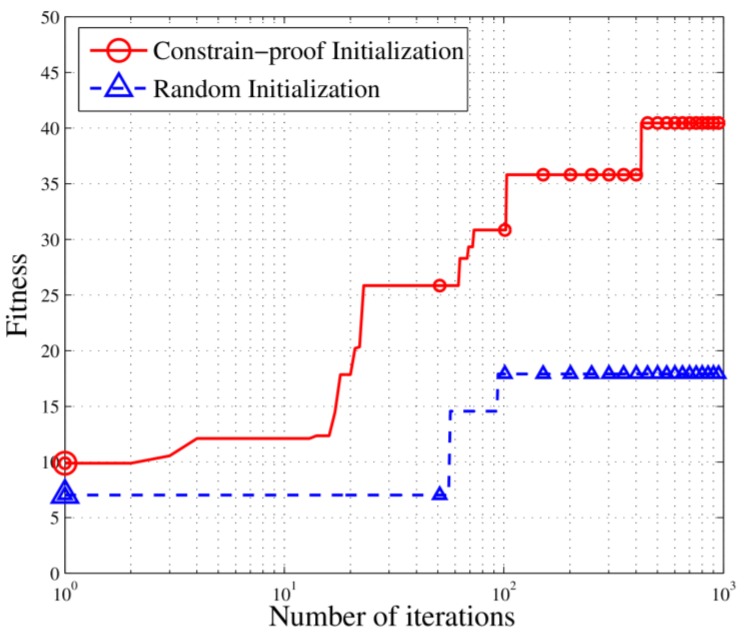
Fitness curves for CI and random initializations

CI has an advantage with respect to astringency because it can provide a specific evolution direction at the beginning of the iterations. Random initialization means the random generation of initial solutions which directly participate in the next operation of the genetic algorithm without checking constraints. In this experiment, in order to compare the difference between CI initialization and random initialization, the parameters are set the same as Table 1 except for the initialization step. And the experiment results are shown in Figure 10.

As shown in Figure 10, the random initialization method fails to find the direction of astringency because it is difficult for random initialization to find an initial value satisfying the constraints and it is also difficult to achieve a continuous reduction of the optimization goals. The CI can accumulate enough solutions to satisfy the constraints in the initialization so that the algorithm can quickly find the appropriate updating direction in the feasible solution space, thus maintaining a considerable descent speed for the optimization target. The results prove that CI can effectively improve the convergence rate of the algorithm.

**Figure 11:**
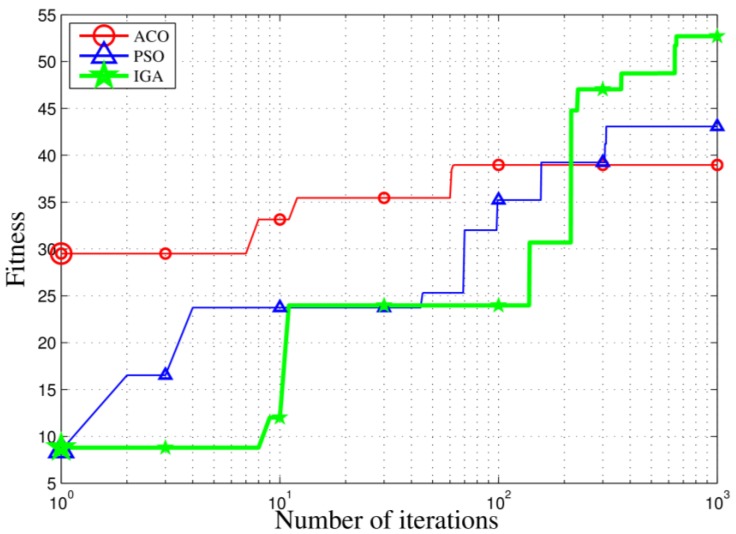
Comparison of different algorithms.

In order to better prove the advantage of IGA algorithm, we conduct a contrast experiment between IGA, particle swarm optimization (PSO) and ant colony optimization (ACO). A simulation with 6 tasks and 25 stations was set up and the parameter settings of PSO and ACO are shown in Table 5. In the scenario, the station frequency of all sites could match the communication frequency of the task, the maximum working capacity of the site was one and the cooperative number was two. The three algorithms were recorded over 1000 iterations to optimize the change of targets. As shown in Figure 11, IGA has a stronger global search ability than ACO and PSO and it can resist the problem of falling into locally optimal points to a certain extent.

#### 5.3.2. Time Complexity

Our requirements for time complexity are relatively high, because location tasks require very strict real-time performance, which means low time delay for processing tasks.

1. Effect of population.

**Figure 12:**
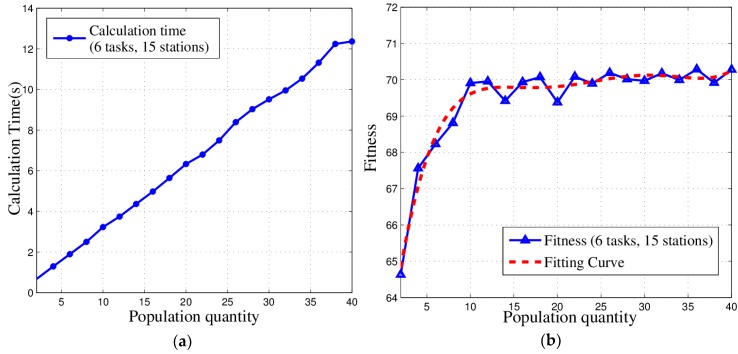
Relationships among time complexity, fitness and population: (**a**) Time complexity-population curve; (**b**) Fitness-population curve.

To investigate the influence of population on time complexity, we built a scenario with 15 stations and six tasks and vary the population from 2 to 40. We examined the time spent for calculation and the fitness of the final solution. From the results of the experiment shown in Figure 12, we can draw the following conclusions. (a) The time of calculation increase approximately linearly according to the population. (b) The fitness can be obviously enhanced by a bigger population at the beginning but when the population is larger than 10, the fitness is no longer sensitive. From this, we can make a final deduction: the population should not increase or decrease without limit and it should be appropriately chosen to balance low time complexity with good global search capability according to real application scenarios.

2. Effect of coding length.

**Figure 13:**
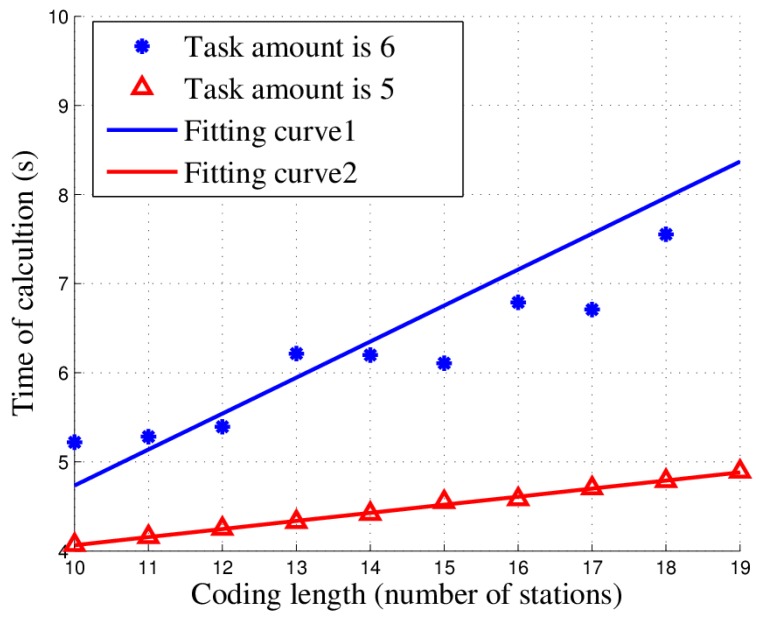
Relationship between time complexity and coding length.

To study the effect of coding length, also regarded as the number of stations, on time complexity, we conducted an experiment to observe the time spent on calculation in different scenarios with different coding lengths. However, the results were not significant because the delay caused by retrogression can enormously impact the final output and obscure the impact of the coding length. Hence, to avoid the retrogression and emphasize the influence of coding length, we fixed the values of the population, maximum number of iteration, frequency, task cooperation requirement and station capabilities. In addition, we varied the coding length from 10 to 19. The relationship between time complexity and coding length becomes more observable, as shown in Figure 13 (two experiments with five and six tasks were conducted).

Clearly, the time of calculation has an approximately linear relationship with the coding length. Moreover, because the coding length may change because of station network adjustments such as added or removed nodes, this experiment demonstrates that the algorithm can maintain a stable time complexity even in rapidly transforming scenarios such as war and this will not lead to a saltation. In conclusion, the algorithm has good time complexity stability in practice.

#### 5.3.3. Location Accuracy

This section discusses the variation tendency of location accuracy during the iterations, which is very important in location tasks. We calculate the PDOP by Equations (3) and (4) and the measurement error Δ is calculated as follows:

(20) $$\Delta =\sum_{i-1}^{N_{t}}\sqrt{{\left({\hat{x}}_{i}-x_{i}\right)}^{2}+{\left({\hat{y}}_{i}-y_{i}\right)}^{2}}$$

Here, ${\hat{x}}_{i}$ and ${\hat{y}}_{i}$ are the measured coordinates of the targets while $x_{i}$ and $y_{i}$ are the real values, respectively.

**Figure 14:**
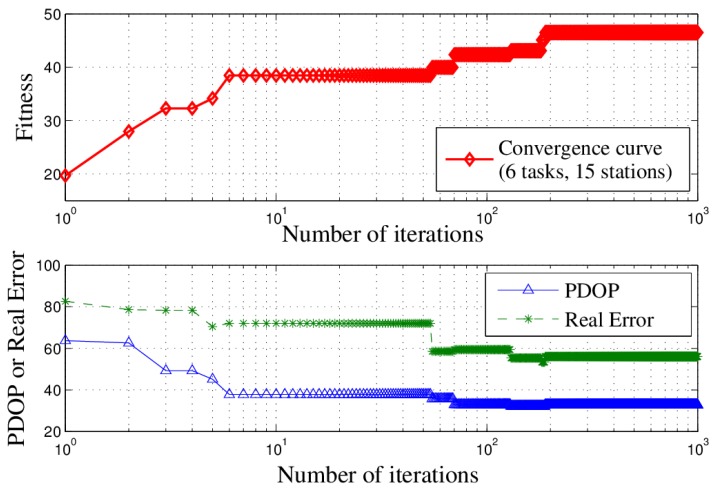
Accuracy over iterations (with six tasks and 15 stations).

The behaviors of Δ and PDOP during the iterations are shown in Figure 14. Generally, as the fitness increases during the iterations, the PDOP value and real measurement error of the solution decrease, indicating that the location accuracy increases. As shown in Figure 14, as the number of iterations increases, the algorithm can improve the location accuracy under the condition of satisfying multiple constraints and completing multiple tasks.

### 5.4. Comparison with the Manual Method

**Figure 15:**
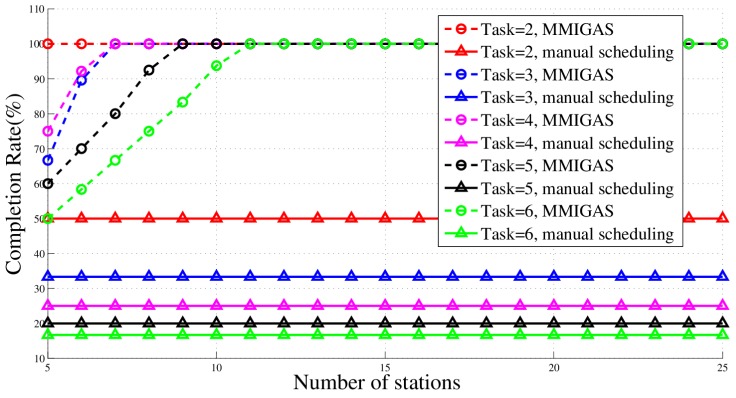
Completion rate in different scenario.

**Figure 16:**
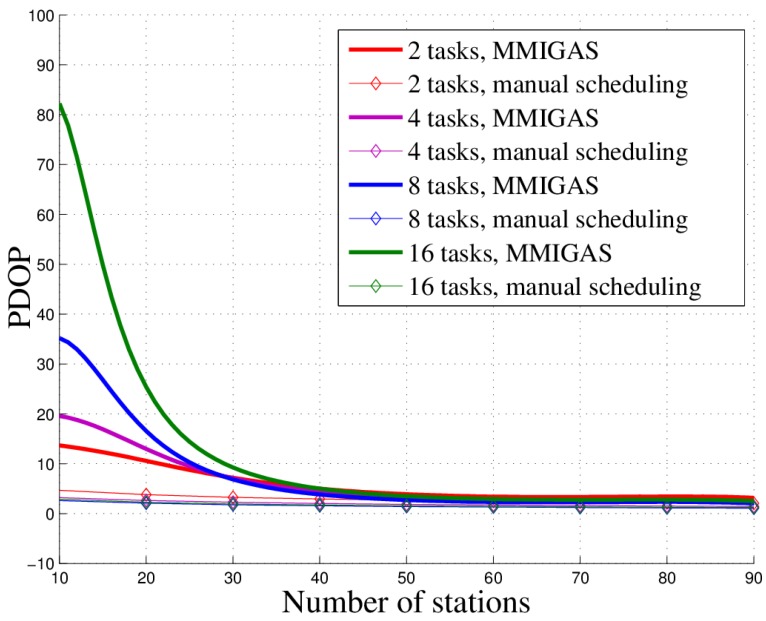
Position dilution of precision (PDOP) in different scenario.

We compare the proposed method and traditional manual scheduling. Here, to simulate the manual method, all stations select the same randomly allocated task for one slot; that is, the task itself is randomly allocated but all stations perform the same one. We used two metrics to examine their performance, PDOP and task completion rate, to indicate the location accuracy and efficiency, respectively. The results, shown in Figure 15 and Figure 16, illustrate two preliminary conclusions. First, MMIGAS has the same location accuracy as the manual scheduling in a scenario with sufficient resources. When the station number is small, the PDOP of manual scheduling is lower than that of MMIGAS because the manual scheduling only finishes one task every slot. However, the gap between them gets smaller and finally goes to zero as the number of stations increases. The proposed method and manual scheduling reach the same accuracy when the number of stations is equal to or more than 60. Second, MMIGAS has a higher completion rate than manual scheduling, which indicates better efficiency. This result also shows that the competition rate grows when the number of stations increases. Eventually, we draw our final conclusion: when the number of stations is high enough, MMIGAS can effectively enhance the efficiency with just a little or even no loss of location accuracy.

For further discussion, we define two values: $Q_{1}$ and $Q_{2}$, which are respectively calculated using (21) and (22).

(21) $$Q_{1}=\frac{p_{1}-p_{2}}{\max\left(p\right)}$$

Here, $p_{1}$ and $p_{2}$ are the PDOP values of MMIGAS and manual scheduling respectively. The physical meaning of $Q_{1}$ is the relative loss of accuracy.

(22) $$Q_{2}=R_{1}-R_{2}$$

Here $R_{1}$ and $R_{2}$ are the completion rates of MMIGAS and manual scheduling respectively. The physical meaning of $Q_{2}$ is the improvement in completion rate.

**Figure 17:**
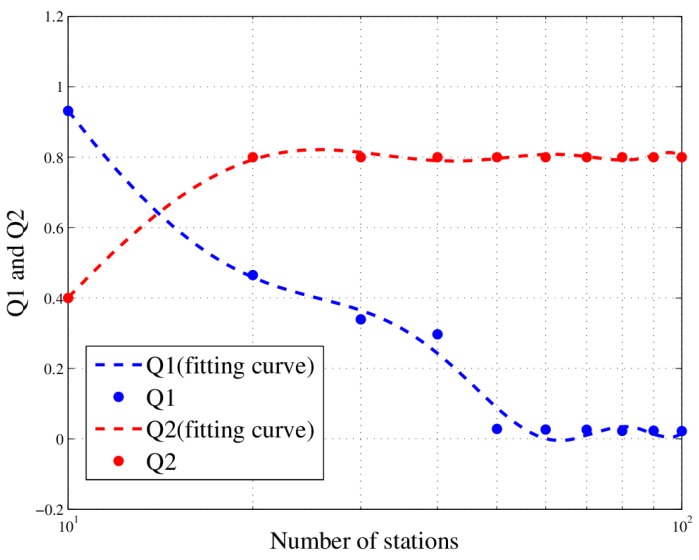
$Q_{1}$ and $Q_{2}$ in different scenario (with 5 tasks).

For five tasks, the curve of $Q_{1}$ and $Q_{2}$ are shown in Figure 17. Clearly, as the number of stations increase, $Q_{1}$ decreases nearly to zero and $Q_{2}$ increases nearly to one, showing that the loss of location accuracy is cut down while the efficiency is significantly improved. Hence, the following can be concluded: when the number of stations is high enough, this method can substantially enhance efficiency at a little cost of location accuracy.

### 5.5. Multi-Objective Function Weights

According to Equation (13), $c_{1}$, $c_{2}$ and $c_{3}$ respectively represent the weights of completion rate, resource utilization and task priority. In Section 5.1, we mainly considered the location accuracy and task completion rate, which mainly aims at accomplishing as many tasks as possible while ensuring accuracy, so the weights are set as $c_{1}=1$, $c_{2}=c_{3}=0$. Then, we will set different values for $c_{1}$, $c_{2}$ and $c_{3}$ to verify the effectiveness of the proposed algorithm for multi-objective situations.

#### 5.5.1. Analysis of Multi-Objective Weights

**Figure 18:**
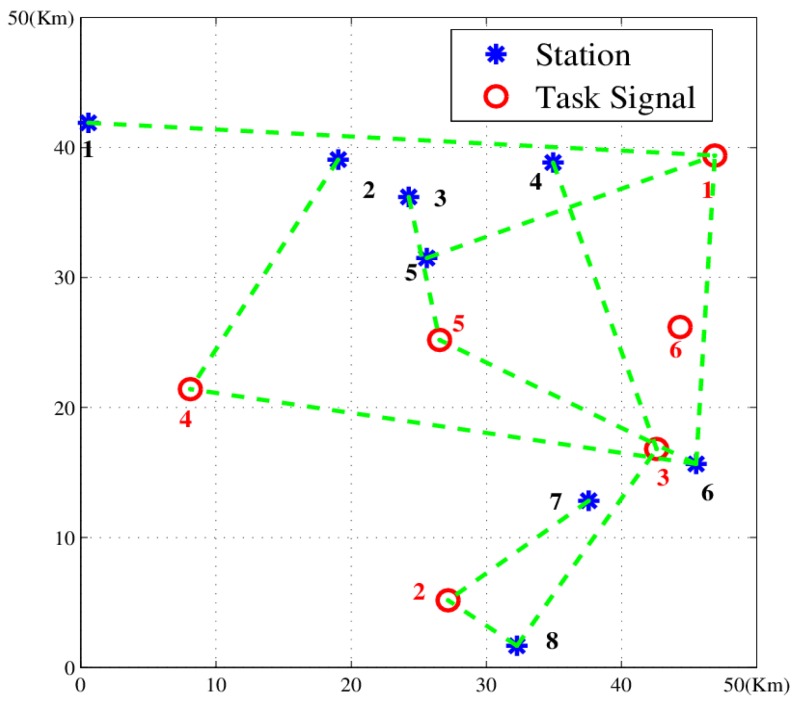
Experiment results of eight stations and six tasks.

First, we set the parameters as: $c_{1}=c_{2}=0.1$, $c_{3}=0.8$. This setting is primarily about task priority and the simulation scenario is to prioritize important tasks when the stations are insufficient. We built up a scenario with eight stations and six tasks. The parameters settings for the algorithm are shown in Table 6, the station scenario parameters are set as shown in Table 2 and the task scenario parameters are set as shown in Table 3. The experiment results are shown in Table 7 and Figure 18.

As can be known from Table 7 and Figure 18, when the number of sites is insufficient, No.6 task is abandoned due to the lowest priority and No.1 task has the most stations and best location accuracy due to the highest priority.

**Figure 19:**
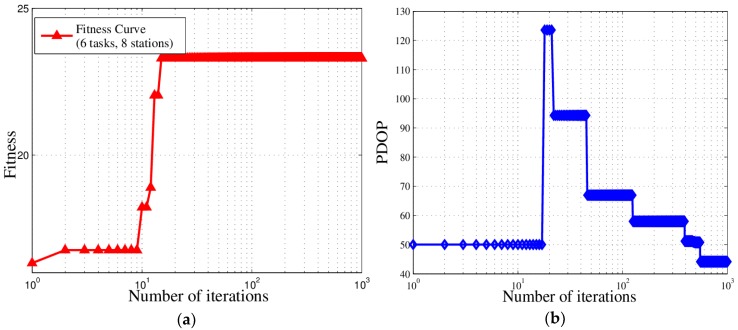

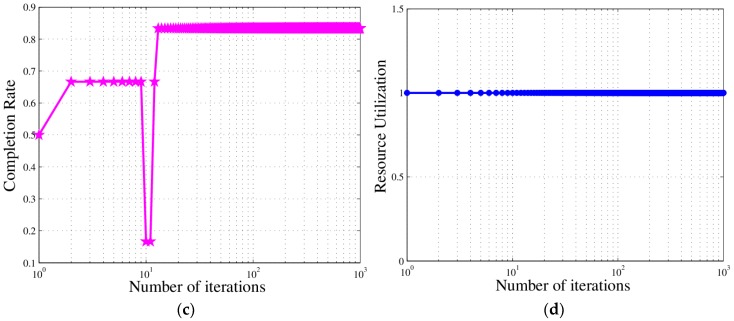
The experiment results in the scenario with 8 stations and 6 tasks ($c_{1}=c_{2}=0.1$, $c_{3}=0.8$): (**a**) fitness curve; (**b**) PDOP curve; (**c**) completion rate curve; (**d**) resource utilization curve.

To analyze the performance of the IGA and verify the experiment conclusions, we adopt the performance and indicators in Section 5.3. And the analysis of experiment results is shown in Figure 19. In Figure 19a, IGA algorithm can still ensure the fitness curve converges to the optimal value. From Figure 19b, we can find out that the PDOP curve is oscillatory due to the influence of priority. And Figure 19c shows that the completion rate did not reach 100% due to insufficient sites and considering the priority, the completion rate curve also oscillated in the early stage. It can be concluded from Figure 19d that because stations are not sufficient, the resource utilization is always at 100%.

Through the experiment analysis, it can be concluded that when the stations are insufficient, MMIGAS can give the optimal solution to different requirements.

#### 5.5.2. Reliability in Different Scenarios

Then, we will combine location accuracy, completion rate, resource utilization and task priority to test the proposed algorithm in different scenarios. Therefore, the task priority is set as Table 7, the parameters are set as $c_{1}=c_{2}=c_{3}=\frac{1}{3}$ and the parameters settings for the algorithm are shown in Table 8. The station and task scenario parameters are also set in Table 2 and Table 3. The experiment results in accordance with the above experimental settings are as follows.

**Figure 20:**
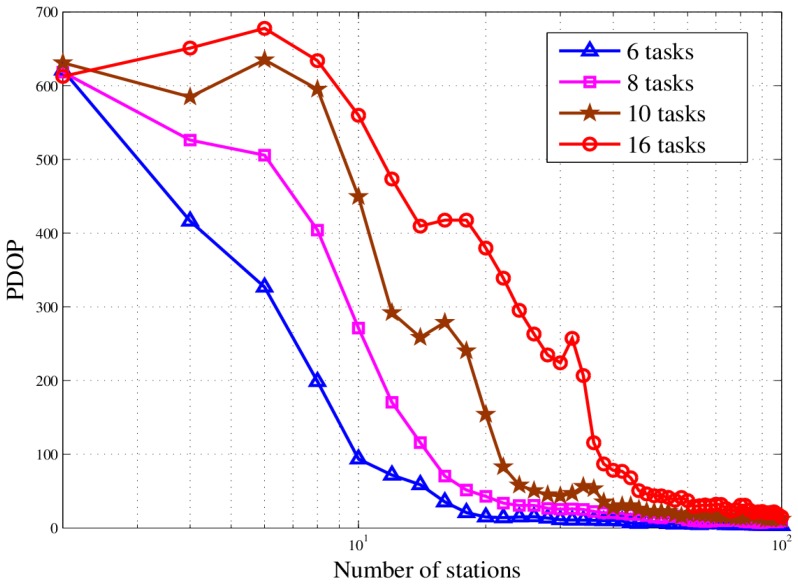
PDOP curve in different scenarios.

From Figure 20, it can be found out that as the number of stations increases, the location accuracy gradually increases. But the station redundancy will occur later. The results show that excessive stations will not bring a noticeable increase in location accuracy when the station number is enough.

**Figure 21:**
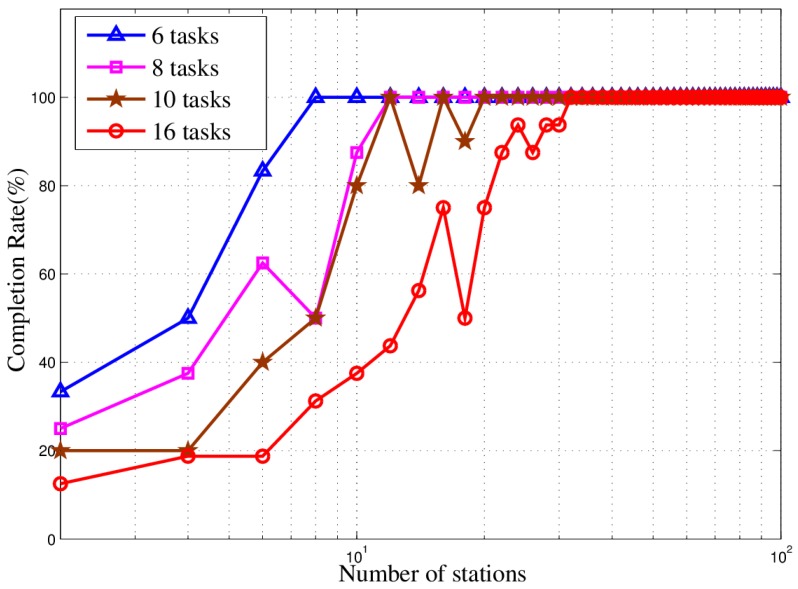
Completion rate curve in different scenarios.

In Figure 21, as the number of stations increases, the task completion rate gradually increases until 100%. Similarly, as the number of tasks increases, so does the number of stations needed to complete all tasks.

**Figure 22:**
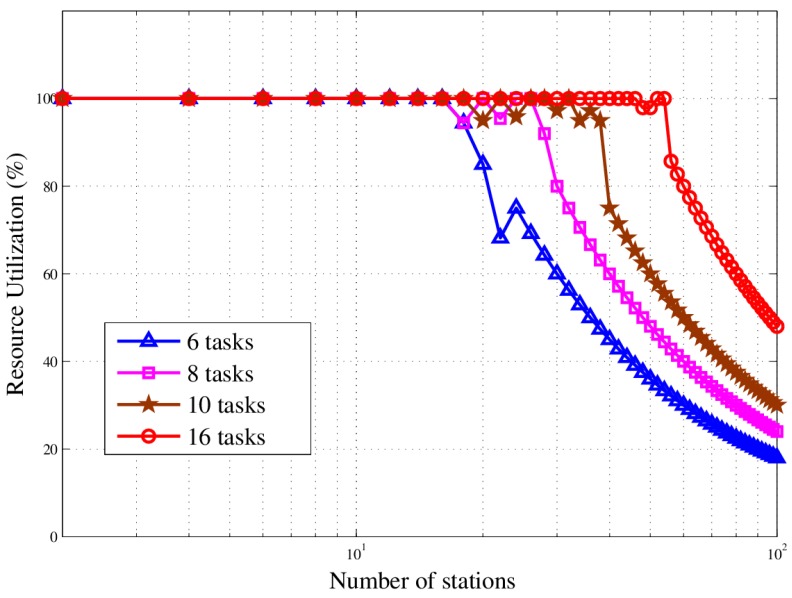
Resource utilization curve in different scenarios.

Figure 22 shows that the utilization rate is 100% in the early stage due to the lack of sites. However, with the increase in the number of stations, station redundancy appears and the resource utilization has gradually declined in all scenarios.

From the above experiment results, we can draw the following two conclusions. First, the algorithm proposed in this paper can well solve the multi-objective and multi-constraint resource scheduling problem of passive location. Secondly, the proposed algorithm has high reliability in different scenarios.

## 6. Conclusions

In this paper, we studied the scheduling problem for passive location resources and proposed a new solution method named MMIGAS. A multi-objective function was conceived to balance various aspects of the performance such as location accuracy, efficiency and resource utilization. And multi-constraint (i.e., frequency, cooperation requirement and capability) was formulated for the practicability of the approach. CI, PF and MGO were implemented to improve the GA, by enhancing the convergence rate and global optimal ability. We evaluated the performance of MMIGAS by computer simulations. In various scenarios, this method can reach its convergence bound within 100 iterations, showing a remarkable astringency. Moreover, its steady time complexity was illustrated because the calculation time was shown to have an approximately linear relationship with population and coding length. Furthermore, we compared traditional manual scheduling and MMIGAS. The results showed that the efficiency is clearly improved by MMIGAS and the location accuracy is still maintained at a high level in a scenario of more than 50 resources at the same time. At last, we also conducted a multi-objective parameter adjustment experiment to verify that the algorithm is suitable for different target requirements and reliable in different scenarios. In conclusion, the proposed algorithm can solve the problem of scheduling of station resources in passive location while maintaining high efficiency and stable location accuracy.

## Figures and Tables

**Table 1:** Parameter settings for the algorithm.

Chromosome Crossover Probability	Individual Crossover Probability	Mutation Probability	Population	Iteration	Penalty Factor	Objective Function Weights	Probability Distribution
0.8	0.8	0.1	10	1000	Ω=5	$c_{1}=1$ $c_{2}=c_{3}=0$	Gaussian N(0,1)

**Table 2:** Parameter settings for the station scenario.

Location Area (km × km)	Beginning Frequency (HZ)	Band (HZ)	Capacity	Probability Distribution
50 × 50	1000~5000	2000~3000	1~2	Uniform Distribution

**Table 3:** Parameter settings for the task scenario.

Location Area (km × km)	Beginning Frequency (HZ)	Band (HZ)	Cooperation Requirement	Probability Distribution
50 × 50	1000~5000	2000~3000	2~3	Uniform Distribution

**Table 4:** Scheduling results of 15 stations and six tasks.

Task	1	2	3	4	5	6
**Station**	1, 3, 14	2, 3, 9	5, 6, 10	8, 12, 13	2, 4, 11	6, 7, 9

Notes: the numbers of task and station are shown in Figure 8.

**Table 5:** Parameter settings for PSO and ACO.

Algorithm	Maximum Iterations	Population	Search Parameters	Other Parameters
PSO	1000	10	$\gamma_{1}=2$, $\gamma_{2}=2$	$V_{\max}=10$
ACO	1000	10	α=2, β=3	η=0.3, cons=10

Notes: In PSO, $\gamma_{1}$ and $\gamma_{2}$ represent the global optimal factor and local optimal factor of particle velocity update in iteration and $V_{\max}$ denotes the maximum velocity of a particle. In ACO, α and β respectively represent pheromone factor and elicitation factor, η denotes pheromone volatilization factor and cons denotes the total amount of pheromone released by an ant at one iteration.

**Table 6:** Parameter settings for the algorithm.

Chromosome Crossover Probability	Individual Crossover Probability	Mutation Probability	Population	Iteration	Penalty Factor	Objective Function Weights	Probability Distribution
0.8	0.8	0.1	10	1000	Ω=5	$c_{1}=c_{2}=0.1$ $c_{3}=0.8$	Gaussian N(0,1)

**Table 7:** Scheduling results of eight stations and six tasks.

Task	1	2	3	4	5	6
**Priority**	6	5	4	3	2	1
**Station**	1, 5, 6	7, 8	4, 8	2, 6	2, 6	——

**Table 8:** Parameter settings for the algorithm.

Chromosome Crossover Probability	Individual Crossover Probability	Mutation Probability	Population	Iteration	Penalty Factor	Objective Function Weights	Probability Distribution
0.8	0.8	0.1	10	1000	Ω=5	$c_{1}=c_{2}=c_{3}=\frac{1}{3}$	Gaussian N(0,1)
